# Cauda Equina Neuroendocrine Tumor: A Histopathological Case Report

**DOI:** 10.7759/cureus.48427

**Published:** 2023-11-07

**Authors:** Hristo Popov, Pavel S Pavlov, George S Stoyanov

**Affiliations:** 1 General and Clinical Pathology, Forensic Medicine and Deontology, Medical University of Varna, Varna, BGR; 2 Clinical Pathology, Complex Oncology Center, Shumen, BGR

**Keywords:** cauda equina, paraganglioma, ependymoma, spinal tumor, neuroendocrine tumor, neuropathology

## Abstract

Cauda equina neuroendocrine tumors (CENET) are rare neoplastic processes that develop in the cauda equina or filum terminale region of the spinal cord, which in previous incarnations of the World Health Organization (WHO) classification of the central nervous system (CNS) tumors were designated as paragangliomas. The change of terminology was carried out due to the rarity of the condition, its specific place of origin, the non-specific clinical and imaging characteristics with which the tumors present, and differences in biological properties (secretion and progression) as well as some minor differences in immunohistochemical protein expression patterns. Herein, we present a case of a male patient in his sixties who presented to us for a histopathological consultation of a previously excised tumor, which was grossly well-demarcated and connected to a nerve root in the cauda equina region. The tumor presented with histomorphological features of a sharply demarcated, non-infiltrative tumor growing in a nested to pseudopapillary pattern with a highly vascularized, intersecting stroma. Tumor cells were mildly atypical ovoid ones, with eosinophilic cytoplasm, central hyperchromatic nuclei, some with nucleoli, and salt and pepper chromatin. Intersecting stroma was rich in reticulin fibers, and the cell did not express epithelial membrane antigen, excluding the diagnosis of ependymoma as well as glial markers, excluding glial origin. Pan-cytokeratin was focally positive, neuroendocrine markers were diffusely positive, and the proliferative index was low. As such, the diagnosis of CENET, WHO CNS grade 1 was established, and the patient was referred back to the institution at which the surgery was performed for follow-up and further management.

## Introduction

Cauda equina neuroendocrine tumor (CENET), previously referred to as paraganglioma, is a rare central nervous system (CNS) tumor that typically develops as per its name in the cauda equina region or the spinal nerve roots. It is a rare and probably underdiagnosed entry, with medical literature reporting only around 300 cases since the nosological entry was initially described back in the 1970s [1]. However, larger-scale analyses of spinal tumors have revealed that around 3% of histologically verified such tumors fall into this group, supporting the notion of these tumors being underrecognized and reported as other entries [2,3].

Patients affected are typically in their fourth to sixth decades, although cases have been described in nearly all age groups [1,4]. Males are more often affected than females [1,5].

Clinical and presenting symptoms are widely unspecific and include lower back pain, numbness, and paresthesia, with manifest cauda equina syndrome being exceedingly rare [5,6]. Functional tumors that secrete hormones or hormone substances can lead to varied presentations, including hypertension, palpitations, headaches, and others, but these are also exceedingly rare [5,7]. As such, they do not present with symptoms that are clinically different from other tumors developing in the same region, which may sway the clinician in thinking about them.

Neuroradiological findings are again non-specific for the tumor entry with high similarity to significantly more common tumors such as ependymoma and schwannoma [6,8]. Tumors are typically sharply demarcated from surrounding structures and may show cystic changes that can have varying rates of signal intensity, even with gadolinium contrast [6,8].

Histopathological diagnosis requires these tumors to be located in the cauda equina region, be sharply demarcated, and have Zellballen architecture or tumor nests separated by vascular stroma; tumor cells must express neuroendocrine differentiation markers [9,10]. The tumor cells can also express cytokeratin, which poses a challenge for the diagnosis when neuroendocrine tumors are unsuspected [11-13]. Reticulin stains typically underline a rich fiber network separating the tumor's nests, and soluble 100 protein (S100) typically highlights a second tumor cell component, sustentacular cells, which surround the Zellballen chief cell nests of the tumor as well [10,11].

## Case presentation

Herein, we present a histopathological case report of a male patient in his early sixties who presented to our team for a histopathological consultation of an excised spinal tumor. Previous medical history was unremarkable except for mild hypertension under adequate medication control for the past two decades. The initial presenting symptoms of the patient were lower back pain, abdominal pain, and difficulties in defecation. The patient was consulted by an outpatient gastroenterologist, with abdominal ultrasound being unremarkable except for ballooning of the large intestine and mild prostate hyperplasia. The patient was then referred to an outpatient urologist and subsequently scheduled for intestinal endoscopy. Prostate-specific antigen came back within reference values, and the patient was referred for abdominal radiology, with computed tomography revealing a tumor formation at the level of the third and fourth lumbar vertebrae. Subsequent magnetic resonance imaging confirmed the tumor formation in the same area as an isointense tumor, sharply demarcated and suspected to be attached to a nerve root and with a diameter of 1.5 cm. The patient was referred to a neurosurgeon, with the neurological status being unremarkable; however, due to the tumor formation on imaging modalities, the patient was scheduled for spinal surgery.

Intraoperatively, a firm, purple-pink, well-demarcated tumor, which was attached to a nerve root with a diameter of 1.3 cm, was visualized at the level of the third and fourth lumbar vertebrae and completely extirpated. Histopathology revealed a non-infiltrative tumor with nested to pseudopapillary growth but did not place a diagnosis, instead offering differentials such as schwannoma, meningioma, and spinal ependymoma.

After surgery, the patient's clinical symptoms resolved completely within a couple of days, and the postoperative period was uneventful.

**Figure 1:**
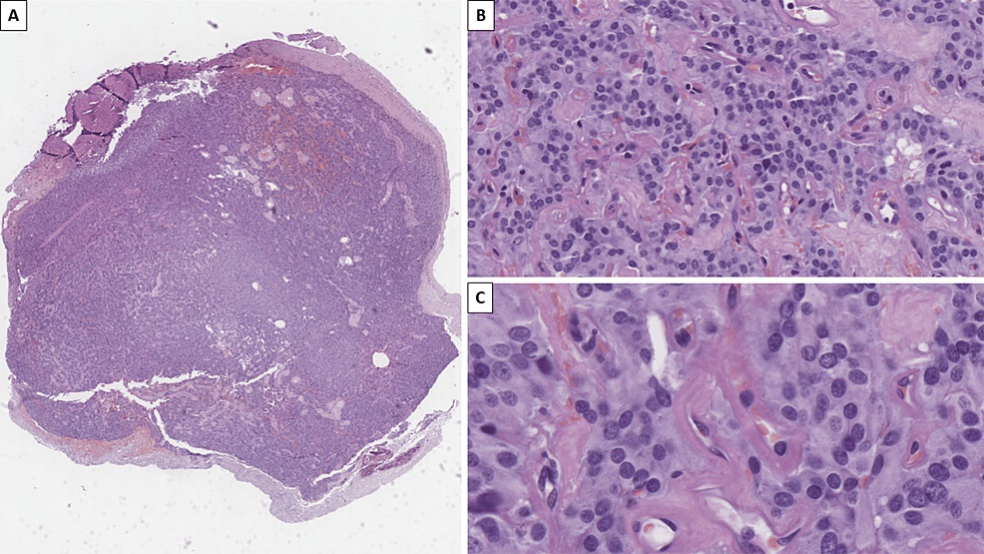
Histopathology of the tumor A: Macro slide view, revealing encapsulation and a non-infiltrative growth, H&E stain. B: Nested to pseudopapillary growth patterns, with intervening vascular stroma; note the cells are ovoid with mild features of atypia, H&E stain, original magnification x200. C: Ovoid cells with brightly eosinophilic to ground-glass opacity cytoplasm, hyperchromatic mildly atypical nuclei, some with nucleoli, and intersecting vascularized stroma, H&E stain, original magnification x400 H&E: hematoxylin and eosin

Histopathology revealed a sharply demarcated, non-infiltrative tumor (Figure 1A). On higher magnification, the tumor cell nests were separated by a highly vascularized, intersecting stroma, with the tumor nests being comprised of mildly atypical ovoid cells, with brightly eosinophilic to ground-glass opacity cytoplasm, central hyperchromatic nuclei, some with nucleoli, and features of salt and pepper chromatin (Figure 1).

**Figure 2:**
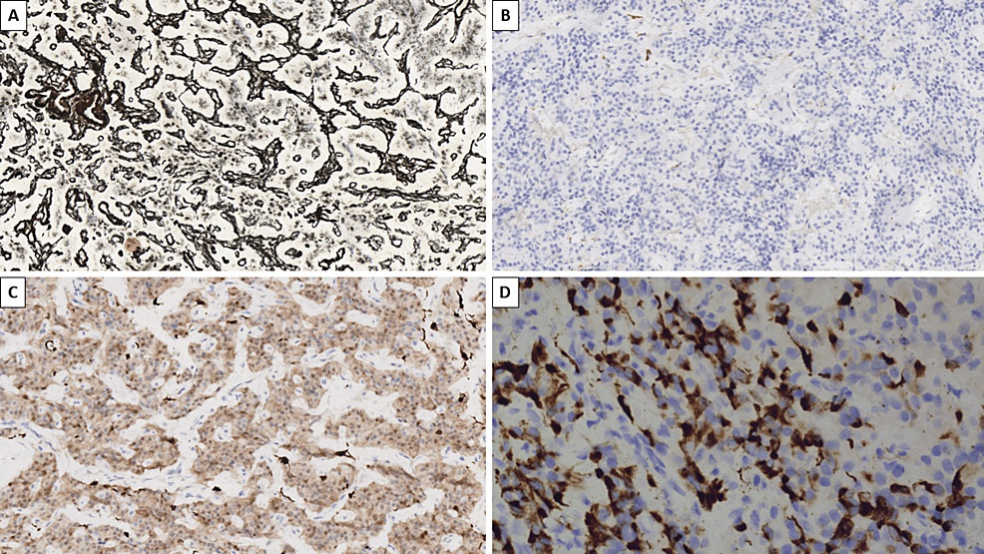
Special stains and immunophenotype of the tumor A: Reticulin fibers intersecting the tumor cell nests, Gomori stain, original magnification x100. B: EMA immunohistochemistry, negative reaction, original magnification x100. C: S100 immunohistochemistry, weak cytoplasmic reaction in the tumor cells, with an intense cytoplasmic reaction in the spindle cells surrounding the nests, original magnification x100. D: CK AE1/AE3 immunohistochemistry, patchy intense cytoplasmic reaction, original magnification x400 EMA: epithelial membrane antigen; S100: soluble 100 protein; CK AE1/AE3: pan-cytokeratin

A classical differential in a tumor with the described morphology is predominantly that of ependymoma; however, based on the extramedullary location, this diagnosis seemed unlikely. Reticulin staining revealed significant fiber deposits intersecting the nests, where the epithelial membrane antigen (EMA), a highly specific marker expressed in ependymoma, did not show positivity, even in a dot-like pattern (Figure 2A and 2B). Immunohistochemistry for S100 revealed a weak cytoplasmic reaction in the tumor cells forming the nests; however, an intense cytoplasmic reaction was noted in spindle cells surrounding the nests (Figure 2C). Pan-cytokeratin (CK AE1/AE3) immunohistochemistry revealed a patchy but intensive cytoplasmic reaction within the tumor cells (Figure 2D).

**Figure 3:**
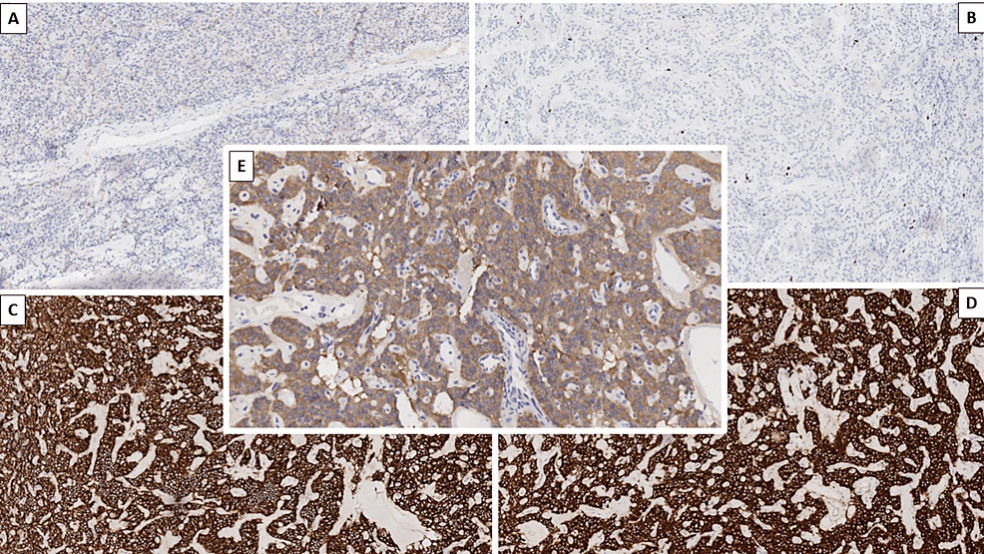
Immunophenotype of the tumor A: Negative GFAP immunohistochemistry, original magnification x100. B: Ki-67 immunohistochemistry, revealing a low proliferative index of around 1%, original magnification x100. C: Diffusely intensive cytoplasmic reaction for CD56, original magnification x100. D: Diffusely intensive cytoplasmic reaction for synaptophysin, original magnification x100. E: Diffusely intermediate cytoplasmic reaction for NSE, original magnification x100 GFAP: glial fibrillary acidic protein; Ki-67: proliferative index; CD56: cluster of differentiation 56; NSE: neuron-specific enolase

The lack of EMA reaction, the pattern of reticulin fiber deposition, and the specifics of the S100 reaction excluded the diagnosis of ependymoma, with glial fibrillary acidic protein reaction coming back negative (Figure 3A) and excluding schwannoma and other gliomas. The proliferative index was low, excluding a metastatic epithelial neoplasm based on pan-cytokeratin staining, while the neuroendocrine marker came back positive (Figure 3B-E).

As per the morphology of the tumor and the spectrum of reaction on special stains and the immunohistochemical profile, the tumor was interpreted as a CENET, specified to be designated in the previous classification as a paraganglioma. As per the World Health Organization (WHO) classification of CNS tumors, the neoplasm was designated a WHO CNS grade 1. The patient was referred to the institution where the surgical procedure was performed for future management.

## Discussion

As mentioned previously, CENET are rare neoplasms. Our case and other similar reports and series show that the principal histopathological differential diagnosis is ependymoma [14]. Despite the clinical history and imaging modalities offering little specific aid in the diagnosis, they remain fundamental in the histopathological differential diagnosis, as CENET are extramedullary tumors attached to a nerve root or the filum terminale, while ependymomas are intramedullary [10]. Furthermore, detailed clinical history and imaging modalities are also helpful in distinguishing metastatic neuroendocrine tumors and neuroendocrine carcinomas to the cauda equina region. While these tumors are rarely well-demarcated, especially in the case of metastatic neuroendocrine carcinomas, there is a more significant degree of cellular and nuclear atypia [15-17].

Clinical history can also suggest the neuroendocrine origin of the tumor in the rare cases when the tumors are secreting. In these instances, intraoperatively, typically, there is a surge in catecholamine excretion from the tumors, and a rapid and significant increase in systemic blood pressure generally is well-recorded [5].

As per the designated WHO CNS grade of 1, CENET are clinically indolent tumors. However, several more extensive series and reports have depicted cases with recurrence and metastatic potential of these rare neoplasms [1,18]. In casuistic cases, when patients present with metastatic spread, differentiation with other neuroendocrine tumors and carcinomas and clinical and histopathological differential diagnosis with other significantly more common sites of origin are difficult, bordering on impossible. In these rare cases, the immunohistochemical marker with the highest reported significance is CK AE1/AE3, which seems consistently positive in CENET and rarely gives a positive reaction in other paraganglioma types of tumors [11-13].

Other, although acceptably rare, cases that may pose a differential diagnosis conundrum are secreting CENET, where the patient presents with catecholamine or other endocrine-related symptoms and the only site detectable is the cauda equina region [1,17]. In such cases, extra CNS origin with metastasis is more probable, as the rate of secreting CENET is low compared to other paraganglioma tumor types and sites of origin [11,17].

As the tumors are designated as WHO CNS grade 1, CENET are typically benevolent tumors, rarely progressing or recurring, except in cases of incomplete excision. Progression with metastatic dissemination is a casuistic feature of these exceedingly rare primary CNS tumors [18].

Case report limitations

Due to the nature of the presented case, mainly the initial symptom analysis and imaging modalities being carried out in an outpatient setting and the surgical procedure, initial histopathological analysis of the tumor, the patient follow-up, and treatment after definitive histopathological diagnosis, our report has several weaknesses. Among these are the lack of correlation between the symptoms and the tumor's location, correlation with the imaging findings on computed tomography and magnetic resonance imaging to the exact location, imaging to gross size, and the comparison of the imaging characteristics and the gross appearance of the tumor itself. As such, the report's primary focus is on the histopathological findings, differential diagnosis, and approach to it. Furthermore, due to the unavailability of direct data from the surgical procedure, its type, possible complications, etc., and the follow-up of the patient at another institution, we cannot provide detailed data on patient recovery parameters and functional outcomes.

## Conclusions

CENET, previously referred to as paragangliomas, are rare primary CNS tumors with neuroendocrine differentiation occurring in the cauda equina and filum terminale region of the spinal cord. The tumors present with non-specific clinical symptoms, and imaging modalities typically offer little specific findings to differentiate them from other tumors common in this area. Histopathological diagnosis is challenging, with the principal differential diagnosis being that of ependymoma and paragangliomas metastatic to this region. As underlined by our case's histopathological findings, a careful approach must be carried out in tumors with such morphology so as to exclude other tumors with similar histopathological findings and apply a detailed panel to confirm nosological origin.
